# Serotype-Specific Differences in the Risk of Dengue Hemorrhagic Fever: An Analysis of Data Collected in Bangkok, Thailand from 1994 to 2006

**DOI:** 10.1371/journal.pntd.0000617

**Published:** 2010-03-02

**Authors:** Jessica R. Fried, Robert V. Gibbons, Siripen Kalayanarooj, Stephen J. Thomas, Anon Srikiatkhachorn, In-Kyu Yoon, Richard G. Jarman, Sharone Green, Alan L. Rothman, Derek A. T. Cummings

**Affiliations:** 1 Mahidol Oxford Tropical Medicine Research Unit (MORU), Faculty of Tropical Medicine, Mahidol University, Bangkok, Thailand; 2 Armed Forces Research Institute of Medical Science (AFRIMS), Bangkok, Thailand; 3 Queen Sirikit National Institute of Child Health, Bangkok, Thailand; 4 University of Massachusetts Medical School, Worcester, Massachusetts, United States of America; 5 Johns Hopkins Bloomberg School of Public Health, Baltimore, Maryland, United States of America; Pediatric Dengue Vaccine Initiative, United States of America

## Abstract

**Background:**

It is unclear whether dengue serotypes differ in their propensity to cause severe disease. We analyzed differences in serotype-specific disease severity in children presenting for medical attention in Bangkok, Thailand.

**Methodology/Principal Findings:**

Prospective studies were conducted from 1994 to 2006. Univariate and multivariate logistic and multinomial logistic regressions were used to determine if dengue hemorrhagic fever (DHF) and signs of severe clinical disease (pleural effusion, ascites, thrombocytopenia, hemoconcentration) were associated with serotype. Crude and adjusted odds ratios were calculated. There were 162 (36%) cases with DENV-1, 102 (23%) with DENV-2, 123 (27%) with DENV-3, and 64 (14%) with DENV-4. There was no significant difference in the rates of DHF by serotype: DENV-2 (43%), DENV-3 (39%), DENV-1 (34%), DENV-4 (31%). DENV-2 was significantly associated with increased odds of DHF grade I compared to DF (OR 2.9 95% CI 1.1, 8.0), when using DENV-1 as the reference. Though not statistically significant, DENV-2 had an increased odds of total DHF and DHF grades II, III, and IV. Secondary serologic response was significantly associated with DHF (OR 6.2) and increased when considering more severe grades of DHF. DENV-2 (9%) and -4 (3%) were significantly less often associated with primary disease than DENV-1 (28%) and -3 (33%). Restricting analysis to secondary cases, we found DENV-2 and DENV-3 to be twice as likely to result in DHF as DEN-4 (p = 0.05). Comparing study years, we found the rate of DHF to be significantly less in 1999, 2000, 2004, and 2005 than in 1994, the study year with the highest percentage of DHF cases, even when controlling for other variables.

**Conclusions/Significance:**

As in other studies, we find secondary disease to be strongly associated with DHF and with more severe grades of DHF. DENV-2 appears to be marginally associated with more severe dengue disease as evidenced by a significant association with DHF grade I when compared to DENV-1. In addition, we found non-significant trends with other grades of DHF. Restricting the analysis to secondary disease we found DENV-2 and -3 to be twice as likely to result in DHF as DEN-4. Differences in severity by study year may suggest that other factors besides serotype play a role in disease severity.

## Author Summary

The four dengue viruses (DENV) represent the most common human arbovirus infections in the world and are currently a challenging problem, particularly in the tropical and subtropical regions of Asia and the Americas. Infection with DENV may produce symptoms of varying severity. While access to care, appropriate interventions, host genetic factors, and previous exposure to DENV are all known to affect the outcome of the infection, it is not entirely understood why some individuals develop more severe disease. It has been hypothesized that the four dengue serotypes differ in disease severity and clinical manifestations. This analysis assessed whether there were significant differences in severity of disease caused by the dengue serotypes in a pediatric population in Thailand. We found significant and non-significant correlations between dengue serotype 2 infection and more severe dengue disease. We also found that individual serotypes varied in disease severity between study years, perhaps supporting the hypothesis that the particular sequences of primary and secondary DENV infections influence disease severity.

## Introduction

Dengue virus (DENV) is an increasing problem in tropical and sub-tropical countries, where *Aedes* spp mosquitoes transmit the virus primarily in urban or semi-urban settings. Infection with DENV may result in a sub-clinical infection, undifferentiated fever, dengue fever (DF), dengue hemorrhagic fever (DHF), or dengue shock syndrome (DSS) [^1^]. Clinical manifestations of DF commonly include fever, rash, hemorrhagic symptoms, headache, ocular pain, arthralgia, myalgia, nausea, and vomiting [^2^]–[^4^]. DHF is difficult to differentiate from DF in the early stages of infection and illness [^4^],[^5^]. The criteria that differentiate DHF from DF are plasma leakage confirmed by pleural effusion, ascites, and/or hemoconcentration (>20% above patient's baseline), and thrombocytopenia (<100,000/mm^3^) [^2^].

While access to care, quality of interventions [^6^], host genetic factors [^7^],[^8^], and previous exposure to DENV [^9^],[^10^] are all known to affect outcome, it is not entirely understood why some individuals develop more severe disease. It has been well established that secondary infections and infections in infants with non-neutralizing maternal antibody to dengue are at increased risk of resulting in DHF [^11^]–[^14^]. Host genetic determinants of disease severity have been reported, including evidence that black patients may have a lower incidence of DHF compared to other patients [^15^]–[^17^]. Some research has indicated that children are more susceptible to developing DHF than adults [^2^],[12],[18],[^19^].

It has been observed that different dengue serotypes, and even strains of the same serotype, differ in their propensity to cause severe disease [^20^],[^21^]. However, there is no clear consensus on the association of DHF and severe disease with serotype. Earlier analyses of data collected during various DF and DHF outbreaks worldwide have shown associations of DENV-2 [^10^], [11], [22]–[^24^] and DENV-3 [^13^],[^21^] with increased risk of hospitalization and severe disease. Small sample sizes, lack of all four serotypes, inconsistencies in findings, and short study durations are weaknesses of these studies. Rico-Hesse *et al*. proposed that virulence of genotypes within serotypes differs, as evidenced by the failure of American DENV-2 in Latin America to produce DHF and the subsequent increase in DHF with the introduction of a Southeast Asian strain of DENV-2 [^20^]. However, neutralization of American DENV-2 by DENV-1 antibodies may have accounted for the absence of DHF in these cases [^25^],[^26^].

We conducted an analysis of data collected at the Queen Sirikit Institute of Child Health (QSNICH) in Bangkok, Thailand to assess whether there were significant differences in severity of disease between the four DENV serotypes in a pediatric population after adjusting for other known risk factors. All four serotypes were encountered, there was consistent application of diagnostic criteria over multiple years, and care was guided by WHO recommendations.

## Methods

### Study Site

A prospective observational study of dengue disease in children at the QSNICH was conducted from 1994–1997, 1999–2002, and 2004–2006 collaboratively by QSNICH, the Armed Forces Research Institute of Medical Science (AFRIMS), and the University of Massachusetts Medical School. QSNICH is a Thai Ministry of Health facility with 538 beds, including a DHF unit with 30 beds and a dengue holding unit with approximately 20 beds. The hospital is a World Health Organization Collaborating Center for Case Management of Dengue.

### Study Population and Data Collection

The clinical study design has been reported previously [^27^]. Briefly, children who presented to the outpatient department or who had already been admitted to the hemorrhagic fever ward were considered for study enrollment based on the following criteria: oral temperature ≥37.5°C or rectal temperature ≥38.5°C; history of fever for <72 hours; no obvious source of infection; weight >6 kg; and age 6 months through 15 years. Exclusion criteria included presentation with clinical signs of shock; or serious chronic disease (thalassemia, nephrosis or nephrotic syndrome, cirrhosis, or malignancy). Due to staffing limitations, only six cases per week could be enrolled. The sampling methods were the same throughout the entire period. We perform all the dengue diagnostic testing for the hospital and found the percentages of each serotype (over the same time period) within 3% of those in the study suggesting no bias in serotype inclusion from the sampling.

Children were monitored as in-patients on the dengue ward, where vital signs and weight were routinely measured. Blood for serologic and clinical studies was drawn each morning for a maximum of five consecutive days while the patient was febrile and on the day following defervescence. A follow-up sample for serological diagnosis was obtained from each child five to ten days after discharge. Plasma leakage was assessed for each patient with chest x-ray, physical exam, and hematocrit changes [^28^]; ultrasound has been studied in a subset of this population, but was not used in this study to classify patients as having ascites, pleural effusion, or DHF. The clinical management of study subjects was directed by the same physician for the study duration.

This study was approved by Human Use Review Committee of the Walter Reed Army Institute of Research, Human Subjects Research Review Board for the Commanding General of the U.S. Army Medical Research and Material Command, University of Massachusetts Medical School IRB, the Thai Ministry of Public Health's Ethical Review Committee for Research in Human Subjects, and the QSNICH IRB. Written informed consent was obtained from the legal guardian of each participant. Secondary data analysis at Mahidol Oxford Tropical Medicine Research Unit and Johns Hopkins University was performed using de-identified data, hence was not deemed human subjects research.

### Definitions

The outcomes of interest were DHF, DF, and DHF severity grades (I–IV). Although outcomes were directed by WHO guidelines, the physician considered the patient's clinical course and treatment in determining final clinical outcomes. Additional outcomes included in the analysis were pleural effusion [measured as both a binary variable and a continuous variable, the pleural effusion index (“PEI” = 100× (maximum width of right pleural effusion)/(maximum width of right hemithorax))] [^28^], ascites, thrombocytopenia, and hemoconcentration. Explanatory variables used were age, sex, study year, blood type, serological response profile (primary vs. secondary infection), and viral serotype. Dengue EIA results were classified as primary if anti-dengue IgM was greater than or equal to 40 units and anti-dengue IgM∶anti-IgG ratio was greater than or equal to 1.8. Results were classified as secondary if anti-dengue IgM was greater than or equal to 40 units and anti-dengue IgM∶anti-IgG ratio was less than 1.8 [^29^].

The case definition for DHF included acute febrile illness; hemorrhagic manifestations (positive tourniquet test, petechiae, epistaxis, gum bleeding or gastrointestinal bleeding); thrombocytopenia (platelet count of <100,000/mm^3^); and plasma leakage evidenced by hemoconcentration ≥20% increase above the patient's baseline, pleural effusion and/or ascites. Cases presenting with the above criteria as well as either narrow pulse pressure (<20 mmHg) or profound hypotension were diagnosed with DSS.

### Laboratory Methods

Clinical tests, diagnostic serology, and virologic tests were performed on all study subjects. These measurements included: complete blood count, hematocrit, ALT, AST, blood group, dengue IgG/IgM antibody, hemagglutination inhibition, dengue RT-PCR [^30^], and virus isolation by mosquito inoculation. Viruses isolated in mosquitoes were serotyped using an antigen capture EIA [^29^]. Differences between the serotype results from RT-PCR and EIA were extremely rare and resolved with repeat testing.

### Statistical Analysis

Only patients with confirmed dengue illness were included in the analysis. Data were entered using FoxPro for Windows software and analysis was performed using Stata/IC 10.0 for Macintosh [^31^]. Crude odds ratios (OR) and their 95% confidence intervals (CI) were calculated. Student's *t* test was used to compare mean values of variables and Pearson's *x* ^2^ test was used to determine significance. Unadjusted and adjusted analyses were performed using univariate and multivariate logistic regression to assess the association of DHF (of any grade) with serotype while controlling for sex, age, blood type, and serological response profile. Multivariate logistic regression was performed by building progressively larger models including Model A (serotype), Model B (serotype, sex), Model C (serotype, sex, age), Model D (serotype, sex, age, primary/secondary infection), and Model E (serotype, sex, age, primary/secondary infection, blood type). Models including interactions between serotypes and all blood types were also assessed [^32^]. Multinomial logistic regression was used to assess the association between the covariates in models A–E and the multinomial outcome of each case as either DF, DHF I, DHF II, DHF III, or DHF IV (5 possible outcomes).

Additional regressions were performed to investigate the association between severe clinical manifestations and serotype. The parameters included in the analysis were pleural effusion/PEI, ascites, hemoconcentration, and plasma leakage, a summary variable considered positive if any of ascites, pleural effusion, or hemoconcentration was present. A two-tailed P-value <0.05 was considered statistically significant.

## Results

### Patient Demographics

**Figure 1:**
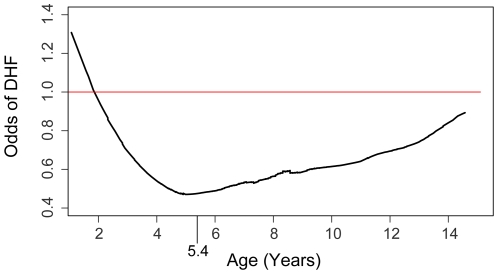
Smoothed lowess plot of odds of DHF vs. age. Odds of DHF upon dengue illness as a function of age (18 months to 15 years). Those not experiencing DHF were diagnosed with DF. A transition point of 5.4 years used to define an indicator variable in regression analyses is shown on the x-axis.

Among the 457 children included in this study, ages ranged from 18 months to 15 years with a mean (SD) age of 8.6 (3.0) years (Table 1). The mean (SD) age of children diagnosed with DF was 8.5 (3.0) years, while the mean (SD) age for children with DHF was 8.7 (3.1) years (p = ns). Based on results from graphical (Figure 1) and exploratory data analyses, we included in our candidate models an indicator variable to control for whether subjects were greater or less than 5.4 years of age. The indicator variable allowed us to estimate different effects of increasing age among those less than 5.4 years of age compared to those older. Figure 1 suggests that, before 5.4 years of age, the risk of DHF declines with increasing age, while after 5.4 years of age the risk increases with increasing age. Males and females were represented with comparable frequency (55.5% male).

**Table 1:** Virological and demographic data by disease severity.

Variable	DF (n = 289)	DHF (n = 169)	p-value	Crude OR for DHF	Adjusted OR for DHF
	n (%)	n (%)		(95% CI)	(95% CI)*
Serotype 1	108 (67)	54 (33)	.	1	1
Serotype 2	58 (57)	44 (43)	0.652	1.5 (0.9, 2.5)	1.2 (0.7, 2.0)
Serotype 3	75 (61)	48 (39)	1	1.3 (.8, 2.1)	1.5 (0.9, 2.4)
Serotype 4	44 (69)	20 (31)	1	0.9 (0.5, 1.7)	0.6 (0.3 1.2)
Male	159 (63)	95 (37)	.	1	1
Female	130 (64)	74 (36)	0.804	1.0 (0.7, 1.4)	1.0 (0.6, 1.4)
Mean age in years (SD)	8.5 (3.0)	8.7 (3.1)	0.382	.	.
age	.	.	.	1.0 (1.0, 1.0)	1.0 (0.9, 1.0)
age>65 months	.	.	.	1.0 (1.0, 1.0)	1.0 (1.0, 1.1)
Primary infection	86 (86)	14 (14)	.	1	1
Secondary infection	202 (57)	155 (43)	<0.001	4.7 (2.6, 8.6)	5.1 (2.7, 9.5)
AB blood type	12 (4)	16 (10)	0.022	2.4 (1.1, 5.2)	3.0 (1.3, 7.0)

*We adjusted for sex, age, blood type, and serological response profile.

### Serological Response Profile

There were statistically significant differences in disease severity among patients with primary and secondary infections. Of the 457 cases with serological data, 14% of patients with primary infections had DHF, whereas 43% with secondary infections had DHF (OR 4.71 95% CI 2.6, 8.6; Table 1). We also found that the severity of DHF was associated with secondary infection. The odds ratio estimated for secondary infection increased with the severity of DHF; secondary infections were more strongly associated with DHF grades III and IV (15.7 95% CI 2.1, 118.3), than either DHF grade I (OR 2.8 95% CI 0.9, 8.5) or DHF grade II (OR 4.9 95% CI 2.3, 10.4).

### Serotypes

DENV-1 was the most commonly found serotype in study subjects, followed by DENV-3, DENV-2, and DENV-4; the eight cases for which viral serotype was unknown were not included in this analysis (Table 2). When adjusting for primary/secondary serological response profile, there was no association found between DHF and serotype (p = 0.295 by Pearson's *x* ^2^ test). When considering a multinomial response (DF, and the four grades of DHF), DENV-2 was found to be statistically significantly associated with increased odds of DHF grade I compared to DF (OR 2.9 95% CI 1.1, 8.0) when compared to DENV-1. None of the DHF grades were associated with DENV-2 when compared to either DENV-3 or DENV-4. Though not statistically significant, DENV-2 was consistently associated with an increased odds of DHF total (OR 1.5 95% CI 0.9, 2.5), DHF grade II (OR 1.3 95% CI 0.7, 2.4) and DHF grades III and IV (OR 1.4 95% CI 0.5, 3.6) compared to DENV-1. Controlling for a primary or secondary serological response, age, season and sex, DHF grade I was again associated with DENV-2 (OR 3.1 95% CI 1.0, 9.4) compared to DENV-1.

**Table 2:** Serotype-specific data.

Year	DENV-1		DENV-2		DENV-3		DENV-4		Totals#		p-value§	p-value##
	DF	DHF	DF	DHF	DF	DHF	DF	DHF	DF	DHF		
	(n = 108)	(n = 54)	(n = 58)	(n = 44)	(n = 75)	(n = 48)	(n = 44)	(n = 20)	(n = 289)	(n = 169)		
	n (%)	n (%)	n (%)	n (%)	n (%)	n (%)	n (%)	n (%)	n (%)	n (%)		
1994	8 (73)	3 (27)	3 (38)	5 (63)	2 (29)	5 (71)	5 (50)	5 (50)	18 (50)	18 (50)	ref	0.02
1995	7 (78)	2 (22)	2 (25)	6 (75)**	5 (71)	2 (29)	0 (0)	1 (100)	14 (56)	11 (44)	NS	0.07
1996	9 (56)	7 (44)	6 (40)	9 (60)	14 (64)	8 (36)	2 (100)	0 (0)	32 (57)	24 (43)	NS	0.05
1997	17 (61)	11 (39)	11 (55)	9 (45)	21 (60)	14 (40)	0 (0)	0 (0)	49 (59)	34 (41)	NS	0.06
1999	8 (80)	2 (20)	2 (67)	1 (33)	14 (78)	4 (22)†	1 (100)	0 (0)	25 (78)	7 (22)	0.02	ref
2000	12 (86)	2 (24)*	6 (75)	2 (25)	4 (80)	1 (20)	2 (67)	1 (33)	24 (77)	7 (23)	0.02	NS
2001	13 (68)	6 (32)	11 (58)	8 (42)	3 (50)	3 (50)	0 (0)	1 (100)	27 (60)	18 (40)	NS	0.09
2002	7 (64)	4 (36)	7 (70)	3 (30)	0 (0)	1 (100)	0 (0)	0 (0)	15 (63)	9 (38)	NS	NS
2004	10 (53)	9 (47)	5 (100)	0 (0)	1 (100)	0 (0)	19 (83)	4 (17)‡	36 (72)	14 (28)	0.04	NS
2005	5 (63)	3 (38)	4 (80)	1 (20)	5 (83)	1 (17)	11 (85)	2 (15)	25 (78)	7 (22)	0.02	ref
2006	12 (71)	5 (29)	1 (100)	0 (0)	6 (40)	9 (60)	4 (40)	6 (60)	24 (55)	20 (46)	NS	0.03
primary	40 (87)	6 (13)	9 (100)	0 (0)	33 (81)	8 (20)	2 (100)	0 (0)	86 (86)	14 (14)	.	.
secondary	68 (59)	48 (41)	48 (52)	44 (48)	42 (51)	40 (49)	42 (68)	20 (32)	202 (57)	155 (43)	.	.

Significant differences between study years within a serotype include:

*: p = 0.05 compared to 2004.

**: p = 0.02 compared to 2004.

**†:** p = 0.03 compared to 2006.

**‡:** p = 0.03 compared to 2006. Significant differences in disease severity between study years overall include:

**§:** most severe study year is used as reference.

## least severe study year is used as reference.

# Discrepancies in totals are due to the 7 cases that were not serotyped and the single case that did not receive a serological response profile.

DENV-1 (28%) and DENV-3 (33%) were more often associated with primary DENV infections than DENV-2 (9%, OR 0.2 95% CI 0.08, 0.5) and DENV-4 (3%, OR 0.06 95% CI 0.01, 0.3). Restricting analysis to secondary cases, we found DENV-2 and DENV-3 to be twice as likely to result in DHF rather than DF compared to DEN-4 (p = 0.05, p = 0.04, respectively).

In aggregate across all serotypes, disease was significantly less severe during 1999 (OR 0.3 95% CI 0.1, 0.8), 2000 (OR 0.2 95% CI 0.1, 0.7), 2004 (OR 0.4 95% CI 0.2, 0.1), and 2005 (OR 0.3 95% CI 0.1,0.8) compared to 1994, the year with the highest percentage of severe cases (Table 2). This is not associated with differences in the serotype distribution in these years compared to others.

As was shown previously with a subset of these data, AB blood type showed a significant association with DHF (OR 2.8 95% CI 1.2, 6.3) [^32^]. There were no significant modification effects when analysis was performed to investigate the interactions between serotypes and blood types.

### Clinical Manifestations

Secondary DENV infection was associated with severe manifestations of pleural effusion/PEI, ascites, and thrombocytopenia; it was not statistically significantly associated with hemoconcentration (Table 3). Univariate analyses showed correlations of both ascites and pleural effusion index (PEI) with DENV-2 (OR 2.1 95% CI 1.0, 4.1; mean PEI was 1.8 times greater for DENV-2; 95% CI 1.1, 3.1); however, multivariate analyses, when controlling for sex, age, study year, and serological response profile, did not (OR 1.5 95% CI 0.7, 3.1; OR 1.5 95% CI 0.8, 2.6). There were no statistically significant associations of the presence of pleural effusion, hemoconcentration, or thrombocytopenia with serotype of infection in univariate or multivariate analyses.

**Table 3:** Clinical manifestations.

Manifestations	DENV-1	DENV-2	DENV-3	DENV-4	Total	Primary Infection	Secondary Infection	Crude OR for Secondary Infection in Patients with vs. without Manifestations
	n (%)	n (%)	n (%)	n (%)	n (%)	n (%)	n (%)	(95% CI, p-value)
Ascites	18 (11*)	21 (21)	15 (12)	9 (14)	63 (14)	5 (8)	58 (92)	3.7 (1.4–9.5, 0.007)
Hemoconcentration	65 (40)	46 (45)	52 (42)	30 (47)	195 (43)	36 (19)	159 (82)	1.4 (0.9–2.3, 0.128)
Thrombocytopenia	99 (61)	62 (61)	74 (60)	34 (53)	274 (60)	36 (13)	238 (87)	3.6 (2.2–5.7, <0.001)
Plasma leakage	86 (53)	63 (62)	72 (59)	36 (57)	261 (57)	46 (18)	215 (82)	1.8 (1.2–2.8, 0.011)
Pleural effusion	55 (34)	41 (40)	45 (37)	23 (36)	167 (37)	15 (9)	152 (91)	4.2 (2.3–7.6, 0.00)
Mean Pleural Effusion Index (PEI)	5.1	8.4†	5	4.3	5.7	0.6	7.1‡	

*Percent of DENV-1 cases with ascites.

†Mean pleural effusion indexes (PEI) were significantly higher among DENV-2 compared to DENV-1.

‡Mean pleural effusion indexes (PEI) were significantly higher among secondary infections as compared to primary infections.

## Discussion

The results of this prospective observational, single-site study of pediatric dengue showed a significant association between DENV-2 and DHF grade I and nonsignificant associations with other grades of DHF. DENV-2 was also associated with the presence of ascites and larger pleural effusions index. Additionally, we confirmed the previously reported associations between secondary serologic response and DHF as well as a more severe grade of DHF.

Other reports have suggested a correlation between serotype and disease severity. Studies in Thailand and Taiwan found a significant correlation between DENV-2 and greater disease severity [^5^],[^9^]. While we found that DENV-2 was most likely to cause DHF (44%) and DENV-4 was less likely (31%) this finding was not statistically significant.

Statistical analysis showed that clinical manifestations of ascites and larger pleural effusions were significantly associated with DENV-2. However, upon adjustment for primary or secondary infection, this association became non-significant. We had limited ability to assess the independent effects of DENV-2 and primary/secondary serological response on the risk of ascites and pleural effusion because such a high percentage of DENV-2 infections were secondary (92%). Several reports have suggested differences in clinical manifestations with distinct dengue serotypes. Vaughn *et al.*, using a subset of data included in this analysis, reported that from 1994–1996, there were more cases of pleural effusion in DENV-2 infections than those infected with the other three serotypes [^9^]. This was not found to be statistically significant in this larger dataset using multivariate analysis incorporating other factors including secondary infection, but the volume of effusions was still greater with DENV-2 as in the earlier study. Additionally, Balmaseda *et al*., found an increased incidence of plasma leakage and thrombocytopenia among those with DENV-1 as compared to DENV-2 infections in Nicaragua from 1999–2001 and during an outbreak in 2003 [^33^]; however, these findings are limited by the short observation period.

Data from this study support the finding that, among those who presented for medical attention, dengue cases caused by DENV-2 and DENV-4 are overwhelmingly secondary infections [^34^], and there were no cases of DHF caused by primary DENV-2 and DENV-4. This suggests that DENV-1 and DENV-3 are more pathogenic without immune priming from other serotypes. It has been reported that DENV-4 causes more mild disease in primary DENV infections [^21]^,[^35^]. We found that DENV-2 and DENV-3 were more likely to cause DHF as secondary infections than DENV-4.

Importantly, this analysis found significantly less severe disease in the study population during 1999, 2000, 2004, and 2005, regardless of viral serotype. (Table 2) Several mechanisms could be responsible for this observation. It is hypothesized that the sequence of serotype infections (both first and second infections) influences disease severity. These years may have been less severe because fewer people had some specific dengue virus immunity that placed them at risk of severe disease upon infection with the predominant viruses circulating those years. It is possible that cross-immunity to viruses circulating in prior years affected disease severity. Finally, there may be significant variation in the risk of severe illness upon infection between viruses within serotypes.

The greatest limitation in this study was that all subjects presented for medical attention, but many who would have been treated as outpatients were admitted for the purposes of this study. By including only patients who seek medical attention, those with milder disease or asymptomatic infections are missed. Additionally, with a limited sample size, estimating models with large numbers of covariates is difficult. Further, only a Thai population was captured; as this study group was ethnographically homogenous, it may not be appropriate to apply the study findings in clinical settings to ethnically diverse populations. Another consideration is that QSNICH is in the urban center of Bangkok and may represent only one of several ecological niches for the dengue vector. However, pediatricians caring for children with suspected dengue infections at the QSNICH are recognized as experts in the management of clinical dengue; consequently, parents from Bangkok and the surrounding area are likely to seek care at QSNICH.

Strengths of the study include the long 11–year data collection period, with virus isolation and serotype identification in 98.5% of cases. This extensive study period, spanning various outbreaks, allowed for the representation of all four serotypes, thus providing data for a more complete analysis. Furthermore, the care and evaluations were performed by the same attending physician throughout the study, reducing the possibility of recording and diagnostic errors and inconsistencies. Lastly, the diagnoses were evaluated in greater depth than standard clinical practice.

In this analysis we found some significant correlations between DENV-2 infection and more severe dengue disease, although other associations were not statistically significant. The increased odds of DHF grade I illnesses among DENV-2 infections compared to DENV-1 is difficult to explain, given we did not observe an increase among higher grades of DHF. While this result might be spurious, it was observed in several models, adjusting for slightly different sets of covariates. Overall, the evidence for DENV-2 infections being more severe is a suggestive trend rather than strong evidence. We did find that individual serotypes varied in disease severity between study years, perhaps supporting the hypothesis that the sequence of serotype infections influences the disease severity.
